# Hybrid Nanofluid Flow Induced by an Oscillating Disk Considering Surface Catalyzed Reaction and Nanoparticles Shape Factor

**DOI:** 10.3390/nano12111794

**Published:** 2022-05-24

**Authors:** Muhammad Ramzan, Saima Riasat, Saleh Fahad Aljurbua, Hassan Ali S. Ghazwani, Omar Mahmoud

**Affiliations:** 1 Department of Computer Science, Bahria University, Islamabad 44000, Pakistan; saimaqau@live.com; 2 Department of Mathematics, College of Science, Qassim University, Buriadah 51452, Saudi Arabia; s.aljurbua@qu.edu.sa; 3 Department of Mechanical Engineering, Faculty of Engineering, Jazan University, Jazan 45124, Saudi Arabia; hghazwani@jazanu.edu.sa; 4 Faculty of Engineering & Technology, Future University in Egypt, New Cairo 11835, Egypt; omar.saad@fue.edu.eg

**Keywords:** heat transfer analysis, hybrid nanofluid, modeling and simulation, numerical solution

## Abstract

Lately, a new class of nanofluids, namely hybrid nanofluids, has been introduced that performs much better compared with the nanofluids when a healthier heat transfer rate is the objective of the study. Heading in the same direction, the present investigation accentuates the unsteady hybrid nanofluid flow involving *CuO*, *Al*_2_*O*_3_/*C*_2_*H*_6_*O*_2_ achieved by an oscillating disk immersed in the porous media. In a study of the homogeneous and heterogeneous reactions, the surface catalyzed reaction was also considered to minimize the reaction time. The shape factors of the nanoparticles were also taken into account, as these play a vital role in assessing the thermal conductivity and heat transfer rate of the system. The assumed model is presented mathematically in the form of partial differential equations. The system is transformed by invoking special similarity transformations. The Keller Box scheme was used to obtain numerical and graphical results. It is inferred that the blade-shaped nanoparticles have the best thermal conductivity that boosts the heat transfer efficiency. The oscillation and surface-catalyzed chemical reactions have opposite impacts on the concentration profile. This analysis also includes a comparison of the proposed model with a published result in a limiting case to check the authenticity of the presented model.

## 1. Introduction

The importance of base fluids (orthodox liquids) for thermal transfer in industrial processes cannot be denied. Generally, these liquids possess poor heat transferability. To overcome this barrier, nano-sized (<100 nm) particles are added to improve the thermal transport capability. This idea was initially pitched by Choi and Eastman [1]. Generally, it is an accepted truth that solid particles have higher thermal heat conduction when compared with liquids. Thus, the inclusion of nano-sized particles in customary fluids improved their thermal conduction comprehensively. These solid particles are identified as nanoparticles. The amalgamation of the base fluid and the nanoparticles is termed a nanofluid. Eastman [2], in an experimental work, claimed that the addition of a small amount of nano-sized solid material particles can improve the thermal conduction of conventional liquids. The conclusion of this study revealed that the thermal performance of ethylene glycol (the base fluid) was improved by 40–50% after the addition of copper nanoparticles or carbon nanotubes (CNTs) at <1% (volume fraction). This is because nanofluids have a pivoting role in electro-mechanical gadgets, heat exchange, advanced cooling systems, etc. A good number of experimental and theoretical studies have been conducted with various combinations of the nanoparticles and base fluids [3,4,5,6,7,8,9,10,11,12].

Lately, an advanced form of nanofluids has been introduced, namely hybrid nanofluids. The hybrid nanofluids comprise an orthodox liquid and two or more kinds of nanoparticles. Hybrid nanofluids are more efficient than customary nanofluids as far as heat transport is concerned. The synthesis of a hybrid nanofluid comprising *Al*_2_*O*_3_-Cu/water using a two-step method was conducted experimentally by Suresh et al. [13]. The study revealed an enhancement of 12.11% in thermal conduction for a 2% volume fraction. Madhesh et al. [14] experimentally discussed a copper–titania hybrid nanofluid and copper–titania hybrid nanocomposite flows with volume concentrations of 0.1–2.0%. The results divulged that the rate of heat flux is improved by 49% for a volume fraction of up to 1%. An experiment was conducted by Toghraie et al. [15] on the synthesis of a ZnO–TiO_2_/EG hybrid nanofluid to demonstrate the impacts of the nanoparticles’ concentration and temperature on the hybrid nanofluid’s conduction. The outcome was interesting, revealing that the thermal conduction was 32% for a volume fraction of 3.5% at 50 °C. Parallel to these experimental works, researchers have also focused on theoretical studies focusing on hybrid nanofluid flows. Gul et al. [16] conducted a comparative study of Hamilton–Crosser, and Yamada–Ota hybrid nanofluid models containing titanium oxide and silicon carbide nanoparticles added into diathermic oil. The hybrid nanofluid was taken through stimulation with a magnetic dipole and the flow was assumed over an extended surface. The salient outcome revealed that the Yamada–Ota model was far better in terms of heat transfer performance than the Hamilton–Crosser hybrid nanofluid flow model. Water-based ternary hybrid nanofluid flows with numerous nanoparticle shapes including spheres, cylinders, and platelets of aluminum oxide, carbon nanotubes, and graphene, respectively, between two parallel sheets, were examined theoretically by Arif et al. [17]. An enhancement in thermal heat transfer of 33.67% was observed for the ternary hybrid nanofluid flow when compared with the unitary nanofluid flow. Recent studies featuring hybrid nanofluid flow in various scenarios may be observed in [18,19,20,21,22].

The problems related to fluid flow over rotating disks are among the well-known active research topics owing to their applicability in many engineering applications encompassing hard disks, jet motors, turbine systems, etc. This is why the subject of rotating flow has gained massive attention and has been welcomed by researchers [23,24,25,26,27,28].

Studies associated with chemical reactions attract the interest of researchers due to their importance in various physical and chemical processes. The molecular diffusion of species in such processes, whether inside or on the surface, cannot be overlooked. Many chemical, biological, and physical processes involve chemical reactions. Therefore, to study these reacting systems, the study of homogeneous and heterogeneous reactions is essential. The presence of a catalyst is essential for a reaction to proceed at a better speed. A reaction occurring on the surface of absorbent media is also a type of heterogeneous reaction and is known as a surface-catalyzed chemical reaction [29]. Elattar et al. [30] computed the hybrid nanofluid flow with Hall current over a slender surface. Recent work focused on chemical reactions may be found in [31,32,33].

The published literature and referenced publications demonstrate a plethora of investigations focusing on nanofluid flows. Nonetheless, there are only a few studies that have shown a comparison of hybrid flows over a variety of geometries. The current study is innovative in several ways: Firstly, the flow over a fluctuating rotating disk was combined with homogeneous–heterogeneous reactions and surface-catalyzed chemical reaction. Secondly, the heat transfer rate was studied, considering the numerous shapes. Thirdly, porous media were incorporated for surface catalysis. To solve the problem, various numerical techniques have been used by various researchers [34,35,36,37,38]. The numerical results were tabulated by using the Keller Box scheme, and the velocity, temperature, and concentration profiles were sketched graphically. Finally, the numerical solution included a validation table to ensure the validity of the proposed model. This research intended to provide answers to the following essential questions:

- Which is the best nanoparticle shape to manufacture a hybrid nanofluid?
- Does a fluctuating rotating disk influence the reaction rate?
- Do the Wall temperature and disk fluctuation both affect the heat transfer rate?
- Is there a significant impact of the surface catalyzed reaction on the rate of reaction?
- Is the axial velocity profile affected by variations in the volume fraction?

## 2. Mathematical Model

**Figure 1:**
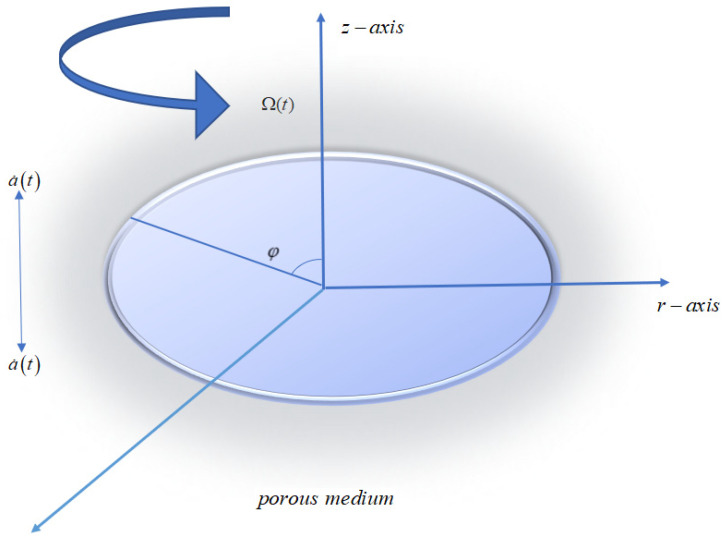
Flow geometry.

Consider an oscillating disk with a velocity $\dot{a}(t)$ immersed in porous media with $CuO,\;Al_{2}O_{3}/C_{2}H_{6}O_{2}$, a hybrid nanofluid with temperature-dependent thermal conductivity. The angular velocity of the disk is Ω(t). The velocity components in the radial, azimuthal, and axial directions are u,v and w. Homogeneous and heterogeneous reactions also occur on the surface of the absorbent media and the disk. The flow diagram is given in Figure 1.

The geometrical and mathematical models drawn under the above assumption are as follows:

The temperature is time-dependent and can be taken in the form [26]:

(1) $$\Delta T=T_{w}(t)-T_{\infty },$$

where $T_{\infty }$ is the ambient fluid temperature and $T_{w}(t)$ is as follows [26]:

(2) $$T_{w}(t)=T_{\infty }+ca{\left(t\right)}^{-2\alpha },$$

The following equation represents the reaction phenomenon [33]:

(3) $$A^{*}+2B^{*}\to 3B^{*},\;\mathrm{rate}=k_{c}C_{a}C_{b}^{2}$$

(4) $$A^{*}\to B^{*},\;\mathrm{rate}=k_{s}C_{a}$$

Under the above assumption, the mathematical model is as described by [33]:

(5) $$\frac{u}{r}+\frac{\partial u}{\partial r}+\frac{\partial w}{\partial z}=0,$$

(6) $$\frac{\partial u}{\partial t}+u\frac{\partial u}{\partial r}+w\frac{\partial u}{\partial z}-\frac{v^{2}}{r}=\frac{1}{\rho_{hnf}}\frac{\partial p}{\partial r}+\frac{\mu_{hnf}}{\rho_{hnf}}\left[\frac{\partial^{2}u}{\partial r^{2}}+\frac{1}{r}\frac{\partial u}{\partial r}+\frac{\partial^{2}u}{\partial z^{2}}-\frac{u}{r^{2}}\right]-\frac{\mu_{hnf}}{\rho_{hnf}}\frac{u}{k^{*}},$$

(7) $$\frac{\partial v}{\partial t}+u\frac{\partial v}{\partial r}+w\frac{\partial v}{\partial z}+\frac{vu}{r}=\frac{\mu_{hnf}}{\rho_{hnf}}\left[\frac{\partial^{2}v}{\partial r^{2}}+\frac{1}{r}\frac{\partial v}{\partial r}+\frac{\partial^{2}v}{\partial z^{2}}-\frac{v}{r^{2}}\right]-\frac{\mu_{hnf}}{\rho_{hnf}}\frac{v}{k^{*}},$$

(8) $$\frac{\partial w}{\partial t}+u\frac{\partial w}{\partial r}+w\frac{\partial w}{\partial z}=-\frac{1}{\rho_{hnf}}\frac{\partial p}{\partial z}+\frac{\mu_{hnf}}{\rho_{hnf}}\left[\frac{\partial^{2}w}{\partial r^{2}}+\frac{1}{r}\frac{\partial w}{\partial r}+\frac{\partial^{2}w}{\partial z^{2}}\right]-\frac{\mu_{hnf}}{\rho_{hnf}}\frac{w}{k^{*}},$$

(9) $${\left(\rho C_{p}\right)}_{hnf}\left(\frac{\partial T}{\partial t}+\frac{\partial T}{\partial r}+u\frac{\partial T}{\partial r}+w\frac{\partial T}{\partial z}\right)=\kappa_{hnf}\left[\frac{\partial^{2}T}{\partial r^{2}}+\frac{1}{r}\frac{\partial T}{\partial r}+\frac{\partial }{\partial z}\left(\frac{\partial T}{\partial z}\right)\right],$$

(10) $$\frac{\partial C_{a}}{\partial t}+u\frac{\partial C_{a}}{\partial r}+w\frac{\partial C_{a}}{\partial z}=D_{A^{*}}\frac{\partial }{\partial z}\left(\frac{\partial C_{a}}{\partial z}\right)-k_{c}C_{a}C_{b}^{2}-\tilde{S}k_{s}C_{a},$$

(11) $$\frac{\partial C_{b}}{\partial t}+u\frac{\partial C_{b}}{\partial r}+w\frac{\partial C_{b}}{\partial z}=D_{B^{*}}\frac{\partial }{\partial z}\left(\frac{\partial C_{b}}{\partial z}\right)+k_{c}C_{a}C_{b}^{2}+\tilde{S}k_{s}C_{a},$$

The mathematical model is subjected to constraints on the boundaries as follows:

(12) $$\begin{array}{c} u=0,v=r\Omega (t),w=0,T=T_{w}(t),D_{A^{*}}\frac{\partial C_{a}}{\partial z}=k_{s}C_{a},D_{B^{*}}\frac{\partial C_{b}}{\partial z}=-k_{s}C_{a}\;\mathrm{at}\;z=0,\\ u\to 0,v\to 0,T\to T_{\infty },C_{a}\to C_{\infty },C_{b}\to 0,\;\mathrm{at}\;z\to \infty , \end{array}$$

We can then apply the following transformation [26]:

(13) $$\begin{array}{l} u=\frac{r\nu }{a^{2}\left(t\right)}f(\eta ),v=\frac{r\nu }{a^{2}\left(t\right)}g(\eta ),w=\frac{\nu }{a\left(t\right)}H(\eta ),p=\frac{\rho \nu^{2}}{a^{2}\left(t\right)}P(\eta ),C_{a}=C_{\infty }\phi ,\\ C_{b}=C_{\infty }\xi ,\theta (\eta )=\frac{T-T_{\infty }}{T_{w}-T_{\infty }},\eta =\frac{z}{a(t)}-1,\eta_{z}=\frac{1}{a(t)},\eta_{t}=-\frac{\dot{a}(t)}{a(t)}\left(\eta +1\right). \end{array}$$

The thermophysical features of the nanoparticles and the base fluid are tabulated in Table 1, and Table 2 presents the sphericity values for numerous nanoparticle shapes.

The thermophysical features in terms of the nanoparticle volume fraction for the hybrid nanofluid are as follows [36]:

(14) $$A=\frac{\mu_{hnf}(T)}{\mu_{f}}={\left(1-\phi_{1}\right)}^{-2.5}{\left(1-\phi_{2}\right)}^{-2.5},$$

(15) $$B=\frac{\rho_{hnf}}{\rho_{f}}=\left(1-\phi_{2}\right)\left\{\left(1-\phi_{1}\right)+\phi_{1}\frac{\rho_{s_{2}}}{\rho_{f}}\right\}+\phi_{2}\frac{\rho_{s_{1}}}{\rho_{f}},$$

(16) $$\begin{array}{l} C_{1}=\frac{k_{hnf}}{k_{bf}}=\frac{k_{s_{2}}-k_{bf}(1-n)+(1-n)\phi_{2}\left(k_{bf}-k_{s_{2}}\right)}{k_{s_{2}}-(1-n)k_{bf}+\phi_{2}\left(k_{bf}-k_{s_{2}}\right)},\\ D=\frac{k_{bf}}{k_{f}}=\frac{k_{s_{1}}-(1-n)k_{f}+(1-n)\phi_{1}\left(k_{f}-k_{s_{1}}\right)}{k_{s_{1}}-(1-n)k_{f}-\phi_{1}\left(k_{f}-k_{s_{1}}\right)},n=\frac{3}{\psi }, \end{array}$$

(17) $$E=\frac{{\left(\rho C_{p}\right)}_{hnf}}{{\left(\rho C_{p}\right)}_{f}}=\left(1-\phi_{2}\right)\left\{\left(1-\phi_{1}\right)+\phi_{1}\frac{{\left(\rho C_{p}\right)}_{s_{1}}}{{\left(\rho C_{p}\right)}_{f}}\right\}+\frac{{\left(\rho C_{p}\right)}_{s_{2}}}{{\left(\rho C_{p}\right)}_{f}}\phi_{2}.$$

The transformed mathematical model is:

(18) 2f′+H=0,

(19) $$f\prime \prime =\left(Hf\prime +f^{2}-g^{2}-S\left[\frac{\eta +1}{2}f\prime +f\right]+\lambda f\right),$$

(20) $$g\prime \prime =\left(Hg\prime +2fg-S\left[\frac{\eta +1}{2}g\prime +g\right]+\lambda g\right)$$

(21) $$\frac{\partial P}{\partial z}=\left(HH\prime -S\left[\frac{\eta +1}{2}H\prime +H\right]+\lambda H\right),$$

(22) $$-\frac{\mathrm{Pr}S}{C_{1}D}\left[\alpha \theta +\frac{\left(\eta +1\right)}{2}\theta \prime \right]+\frac{A}{BC_{1}D}\mathrm{Pr}H\theta \prime =\left(1+\varepsilon \theta \right)\theta \prime \prime +\varepsilon \theta \prime^{2}$$

(23) $$S(\eta +1)\phi \prime -h\phi \prime =-\frac{1}{Sc}\phi \prime \prime +K_{c}\phi {\left(1-\phi \right)}^{2}-K_{vs}\phi ,$$

(24) $$-S(\eta +1)\xi \prime +h\xi \prime =\frac{\delta }{Sc}\xi \prime \prime +K_{c}\phi \xi^{2}+K_{vs}\xi ,$$

The transformed bounded constraints are:

(25) $$\begin{array}{c} f(0)=0,g(0)=\omega ,H(0)=0,\theta (0)=1,\xi \prime (0)=K_{s}\xi (0),\phi \prime (0)=K_{s}\phi (0),\\ f\left(\infty \right)\to 0,g\left(\infty \right)\to 0,\theta (\infty )\to 0,\phi (\infty )\to 1,\xi (\infty )\to 0, \end{array}$$

where $a(t)=d\sqrt{1+\frac{\nu_{hnf}S}{d^{2}}t}$ is the displacement of the oscillatory disk. The rotation parameter is $\omega =\frac{\Omega (t)a^{2}(t)}{\nu_{hnf}}$, and $S=2\frac{a(t)\dot{a}(t)}{\nu_{hnf}}$ is the parameter for controlling the contraction and expansion of the disk.

Assuming both species have comparatively the same size, we have:

(26) δ=1, ξ(η)+ϕ(η)=1,

Equations (23) and (24) will be reduced to:

(27) $$Sc\left[K_{c}\phi {\left(1-\phi \right)}^{2}-S\left(\eta +1\right)\phi \prime +h\phi \prime \right]=\phi \prime \prime ,$$

with the associated boundary condition:

(28) $$\phi \prime \left(0\right)=K_{s}\phi \left(0\right),\phi \left(1\right)\to 1,$$

where:

(29) $$\begin{array}{l} \frac{a^{2}(t)}{k^{*}}=\lambda ,Sc=\frac{\nu_{f}}{D_{A^{*}}},K_{c}=\frac{k_{c}C_{o}^{2}}{\Omega },K_{vs}=S_{v}K_{s},K_{s}=\frac{k_{s}\sqrt{\nu_{f}}}{D_{A^{*}}\sqrt{\Omega }},\\ \delta =\frac{D_{B}}{D_{A}},\mathrm{Pr}=\frac{C_{p}\mu_{f}}{k_{f}}S_{v}=\frac{\tilde{S}D_{A}}{\sqrt{\nu_{f}\Omega }}. \end{array}$$

where $\lambda ,Sc,K_{c},K_{vs},K_{s},\delta ,\mathrm{Pr},S_{v}$ represent the porosity parameter, Schmidt number, homogeneous reaction parameter, surface catalyzed parameter, heterogeneous reaction parameter, the ratio of diffusion coefficient, Prandtl number, and parameter of interfacial area, respectively. Detailed work for the conversion of a system of partial differential equations to a system of ordinary differential equations is given in the Appendix A. 

## 3. Numerical Scheme (Keller Box)

The transformed mathematical model is tackled by using the Keller Box scheme. The numerical procedure involves the following steps.

First, we utilize the following transformation to convert the problem into the first order.

(30) $$f\prime =-\frac{H}{2},g\prime =X,\theta \prime =Y,\phi \prime =Z$$

The transformed mathematical model is as follows:

(31) $$H\prime =2\left(\frac{H^{2}}{2}-f^{2}+g^{2}-S\left[\frac{\eta +1}{4}H-f\right]-\lambda f\right),$$

(32) $$X\prime =\left(HX+2fg-S\left[\frac{\eta +1}{2}X+g\right]+\lambda g\right),$$

(33) $$-\frac{\mathrm{Pr}S}{C_{1}D}\left[\alpha \theta +\frac{\left(\eta +1\right)}{2}Y\right]+\frac{A}{BC_{1}D}\mathrm{Pr}HY=\left(1+\varepsilon \theta \right)\theta Y\prime +\varepsilon Y^{2}$$

(34) $$Sc\left[K_{c}\phi {\left(1-\phi \right)}^{2}-S\left(\eta +1\right)Z+HZ\right]=Z\prime ,$$

The boundary conditions are:

(35) $$\begin{array}{c} f(0)=0,g(0)=\omega ,H(0)=0,\theta (0)=1,\phi \prime (0)=K_{s}\phi (0),\\ f\left(\infty \right)\to 0,g\left(\infty \right)\to 0,\theta (\infty )\to 0,\phi (\infty )\to 1, \end{array}$$

Next, consider the discretization of η the axis with a step size $h_{j}$ such that:

(36) $$\eta_{o}=0,\eta_{j}=h_{j}+\eta_{j-1},j=1,2,3,\ldots J.\mathrm{For}J\to \infty ,\eta_{J}=\eta_{\infty }.$$

For the point $\eta_{j-1/2}$ on the η axis, we have a central difference approximation defined by:

(37) $$\begin{array}{l} f_{j-1/2}=\frac{f_{j}^{i}+f_{j-1}^{i}}{2}\\ f_{{}_{j-1/2}}^{\prime }=\frac{f_{j}^{i}-f_{j-1}^{i}}{h_{j}} \end{array}$$

**Figure 2:**
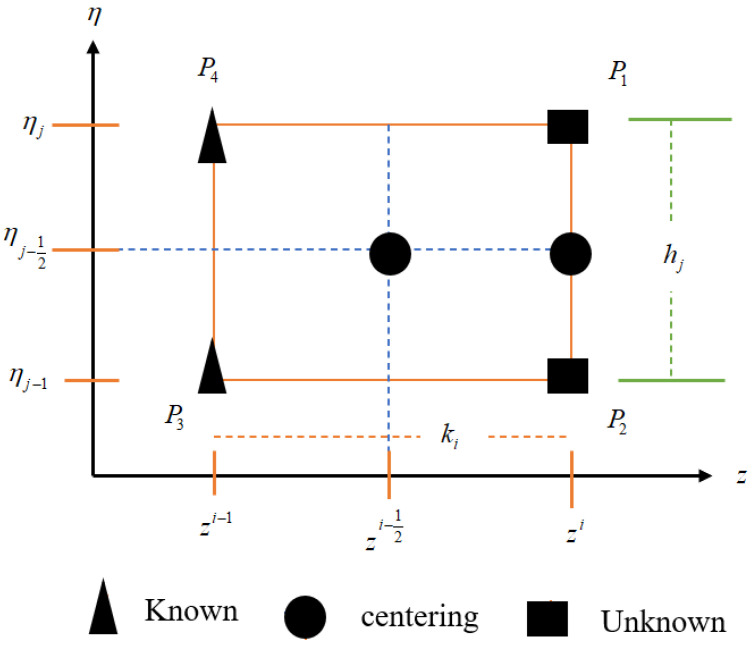
Schematic diagram for domain discretization.

The discretization in the η−z plane is explained in Figure 2.

(38) $$z^{0}=0,\;z^{i}=z^{i-1}+k_{i,\;}i=1,2,3,\ldots I,$$

(39) $$\eta_{0}=0,\;z^{j}=\eta_{j-1}+h_{j,\;}j=1,2,3\ldots J,$$

where $k_{i}(\Delta z)$ and $\;h_{j}(\Delta \eta )$ are the spacing centering on the point $\left(z^{i},\eta_{j-\frac{1}{2}}\right)$:

(40) $$\begin{array}{l} \frac{f_{j}^{i}-f_{j-1}^{i}}{h_{j}}=-\frac{H_{j}^{i}+H_{j-1}^{i}}{4}\\ \frac{g_{j}^{i}-g_{j-1}^{i}}{h_{j}}=\frac{X_{j}^{i}+X_{j-1}^{i}}{2}\\ \frac{\theta_{j}^{i}-\theta_{j-1}^{i}}{h_{j}}=\frac{Y_{j}^{i}+Y_{j-1}^{i}}{2}\\ \frac{\phi_{j}^{i}-\phi_{j-1}^{i}}{h_{j}}=\frac{Z_{j}^{i}+Z_{j-1}^{i}}{2} \end{array}$$

Combining Equation (37) into Equations (31)–(34) and using central difference approximation, we have the following system of equations:

(41) $$L_{1}=\left(\frac{H_{j}^{i}-H_{j-1}^{i}}{h_{j}}\right)-\frac{2B}{A}\left(\begin{array}{l} \frac{{\left(\frac{H_{j}^{i}+H_{j-1}^{i}}{2}\right)}^{2}}{2}-{\left(\frac{f_{j}^{i}+f_{j-1}^{i}}{2}\right)}^{2}+{\left(\frac{g_{j}^{i}+g_{j-1}^{i}}{2}\right)}^{2}\\ -S\left[\frac{\eta +1}{4}\left(\frac{H_{j}^{i}+H_{j-1}^{i}}{2}\right)-\left(\frac{f_{j}^{i}+f_{j-1}^{i}}{2}\right)\right]-\lambda \left(\frac{f_{j}^{i}+f_{j-1}^{i}}{2}\right) \end{array}\right),$$

(42) $$\frac{X_{j}^{i}-X_{j-1}^{i}}{h_{j}}-\frac{B}{A}\left(\begin{array}{l} \frac{H_{j}^{i}+H_{j-1}^{i}}{2}\frac{X_{j}^{i}+X_{j-1}^{i}}{2}+2\frac{f_{j}^{i}+f_{j-1}^{i}}{2}\frac{g_{j}^{i}+g_{j-1}^{i}}{2}-\\ S\left[\left(\frac{\eta +1}{2}\right)\frac{X_{j}^{i}+X_{j-1}^{i}}{2}+\frac{g_{j}^{i}+g_{j-1}^{i}}{2}\right]+\lambda \frac{g_{j}^{i}+g_{j-1}^{i}}{2} \end{array}\right)=L_{2},$$

(43) $$\begin{array}{l} -\frac{\mathrm{Pr}S}{C_{1}D}\left[\alpha \frac{\theta_{j}^{i}+\theta_{j-1}^{i}}{2}+\frac{\left(\eta +1\right)}{2}\frac{Y_{j}^{i}+Y_{j-1}^{i}}{2}\right]+\frac{A}{BC_{1}D}\mathrm{Pr}\frac{H_{j}^{i}+H_{j-1}^{i}}{2}\frac{Y_{j}^{i}+Y_{j-1}^{i}}{2}\\ -\varepsilon {\left(\frac{Y_{j}^{i}+Y_{j-1}^{i}}{2}\right)}^{2}=\left(1+\varepsilon \frac{\theta_{j}^{i}+\theta_{j-1}^{i}}{2}\right)\frac{\theta_{j}^{i}+\theta_{j-1}^{i}}{2}L_{3}, \end{array}$$

(44) $$Sc\left[K_{c}\frac{\phi_{j}^{i}+\phi_{j-1}^{i}}{2}{\left(1-\frac{\phi_{j}^{i}+\phi_{j-1}^{i}}{2}\right)}^{2}-S\left(\eta +1\right)\frac{Z_{j}^{i}+Z_{j-1}^{i}}{2}+\frac{H_{j}^{i}+H_{j-1}^{i}}{2}\frac{Z_{j}^{i}+Z_{j-1}^{i}}{2}\right]=L_{4},$$

The boundary constraints are:

(45) $$\begin{array}{l} f_{0}=0,g_{0}=\omega ,H_{0}=0,\theta_{0}=1,Z_{0}=K_{s}\phi_{0},\\ f_{J}\to 0,g_{J}\to 0,\theta_{J}\to 0,\phi_{J}\to 1, \end{array}$$

To linearize the system, we utilized Newton’s method for iteration to solve the system of equations above. The block tridiagonal elimination technique was used to solve the system of equations obtained.

## 4. Results and Discussion

This section addresses the numerous parameters’ impact on the associated profiles. The discussion is segmented into subsections.

### 4.1. Thermal Conductivity and Heat Transfer Rate for Different Nanoparticle Shapes

**Figure 3:**
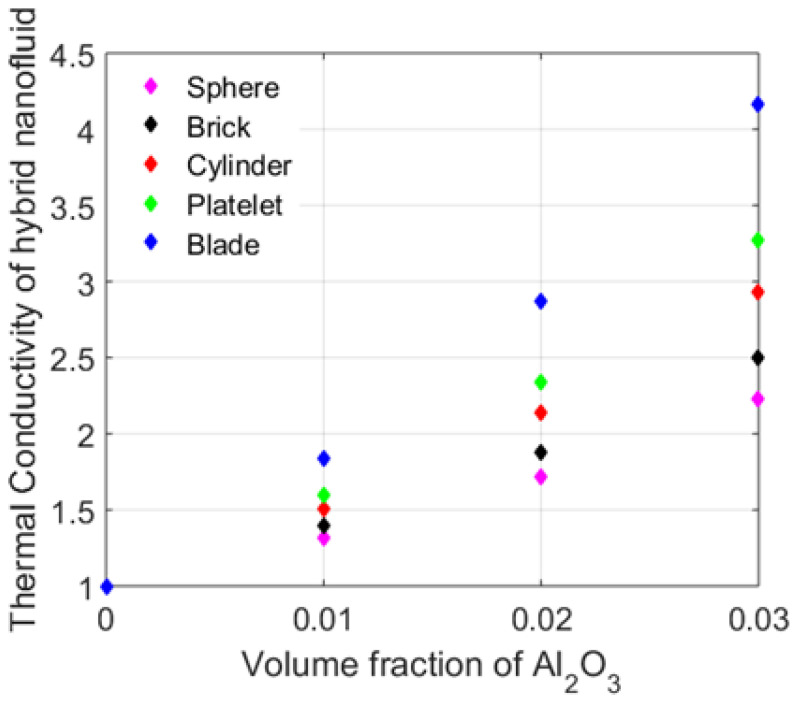
Thermal conductivity of the hybrid nanofluid for various nanoparticle shapes.

**Figure 4:**
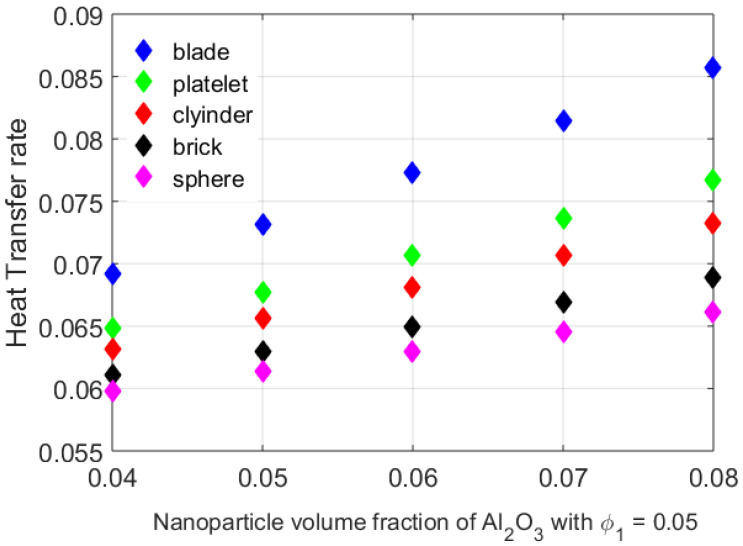
Heat transfer rate for different particle shapes.

Figure 3 addresses the variable thermal conductivity of the $CuO,Al_{2}O_{3}/C_{2}H_{6}O_{2}$ hybrid nanofluid with different shapes and volume fractions of $Al_{2}O_{3}$ by keeping the volume fraction of CuO $\phi_{1}=0.05$. With $\phi_{2}=0.07$, the blade-shaped nanoparticles would give the best thermal conductivity as compared with the other shapes. Figure 4 delineates the heat transfer analysis by taking different values for the volume fraction of $Al_{2}O_{3}$ and considering various nanoparticle shapes. The results obtained were found to have a good correlation with Figure 2. The blade-shaped $Al_{2}O_{3}$ with $\phi_{1}=0.05,\phi_{2}=0.07$ (the volume fraction of CuO) had a higher heat transfer rate. As the Nusselt number is the fraction of the convective heat transfer and the conductive heat transfer, the blade-shaped particles would have the maximum heat transfer rate. However, the heat transfer rate was minimum for the spherical particles.

### 4.2. Velocity Profile versus Varying Parameters

**Figure 5:**
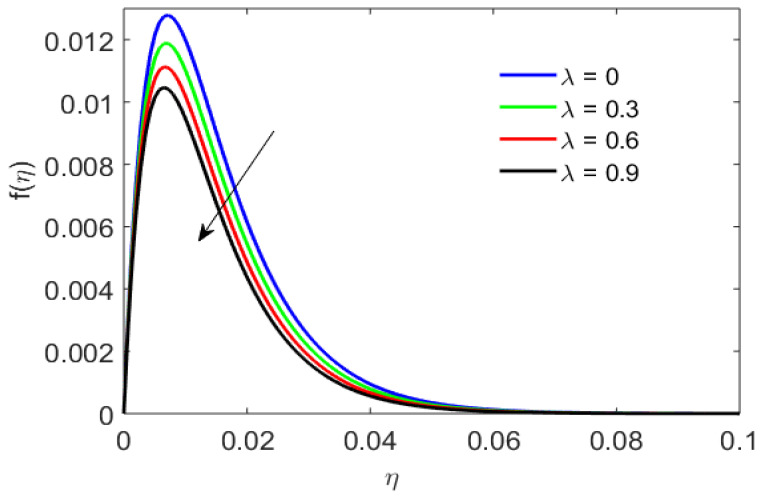
Radial velocity versus porosity parameter.

Figure 5 gives the radial distribution of velocity for an oscillating disk with varying porosity parameters. The porous media provide a large surface area for the fluid particles to penetrate. As the fluid particles penetrate and are absorbed into the pores, causing a deceleration in the particles’ motion. Therefore, the motion of the particles executed by the oscillating disk is inhibited by the presence of porous media. Thus, increasing the value of porosity results in a declining radial profile.

**Figure 6:**
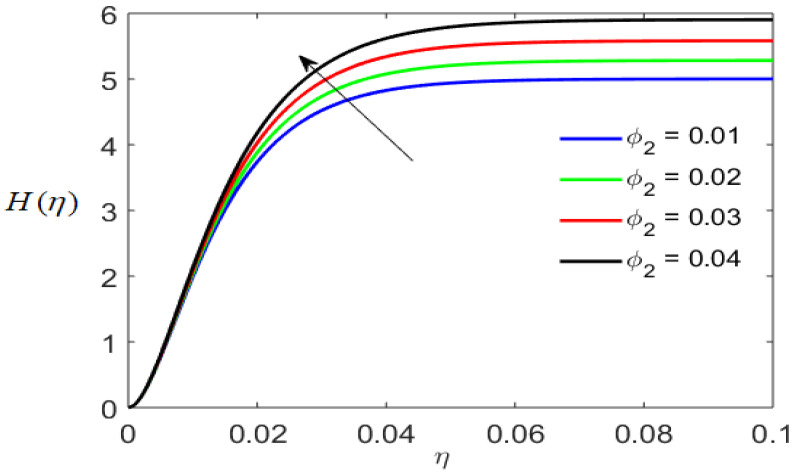
Axial velocity profile for varying volume fractions of nanoparticles of $Al_{2}O_{3}$.

Figure 6 outlines the axial velocity distribution for varying volume fractions of spherical nanoparticles of $Al_{2}O_{3}$. With an increase in the concentration of spherical nanoparticles of $Al_{2}O_{3}$, the axial velocity distribution produces a high curve. Physically, the increase in the volume fraction means that many particles have been added. The momentum transfer process accelerates owing to the enhancement of the axial inflow.

### 4.3. Concentration Profile versus Varying Parameters

**Figure 7:**
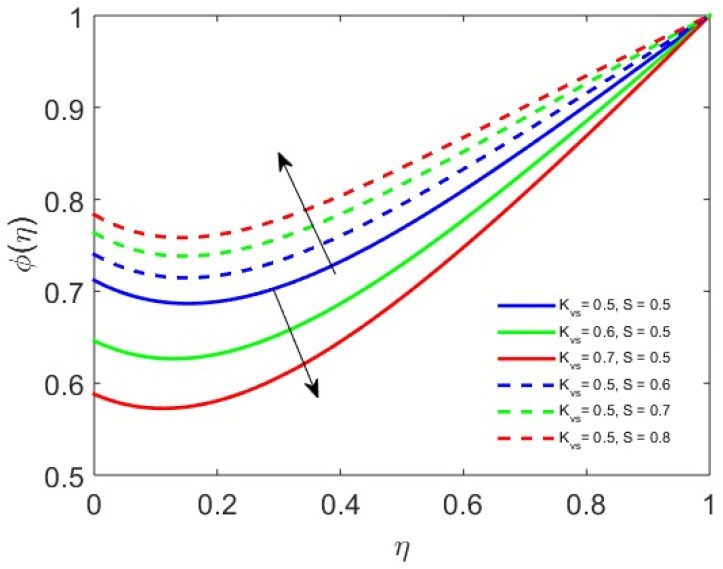
Profile of concentration (ϕ) versus $K_{vs}$ and S.

Figure 7 shows the concentration profile with fluctuations in the surface catalysis and disk parameter. It is noticed that the surface catalysis parameter boosts the reaction rate, causing the concentration profile to decline, while the oscillating disk parameter inhibits the reaction rate at the same rate. Therefore, the concentration profile is increased through contraction and relaxation of the disk.

### 4.4. Temperature Profile versus Varying Parameters

**Figure 8:**
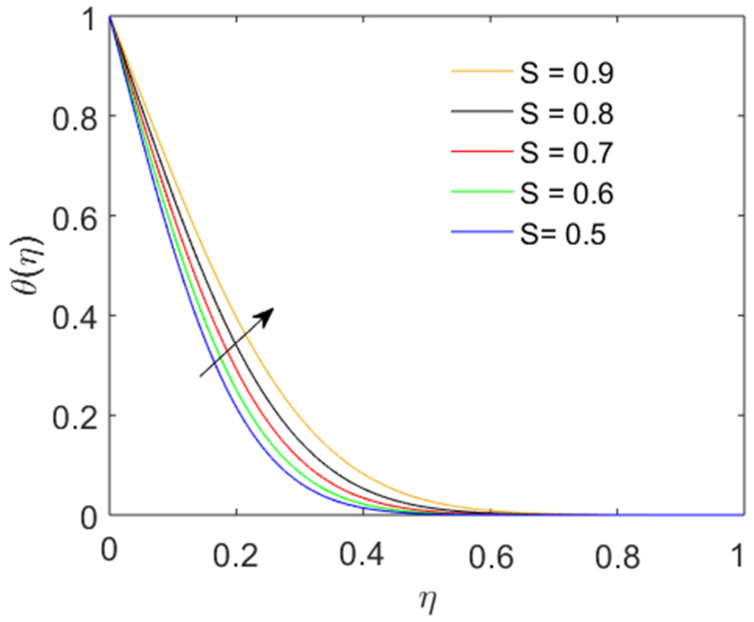
Impact of the disk fluctuation parameter on the thermal profile.

The temperature profile increases with the expanding and contracting disk parameter S in Figure 8. An escalating thermal profile is observed for increasing values of S. As the fluctuating motion of the disk affects the adjacent layers of the fluid, the energy transmission process increases, causing the thermal profile to increase. Near the disk, the thermal amplitude is at its maximum.

### 4.5. Numerical Results for Drag Force and the Heat and Mass Transfer Rate

The numerical results for drag force (Re1/2Cf), the heat transfer rate (Re−1/2Nu), and the mass transfer rate (Re−1/2Sh) are delineated by tabulating the results obtained. Table 3 shows the numerical results for the drag force, heat, and mass transfer rate by fixing $K_{vs}=0.5,\lambda =0.5,\Omega =0.1,Sc=1,\mathrm{Pr}=1,\alpha =0.5,K_{s}=0.5,K_{c}=0.5$ for increasing values of the oscillating parameter S. This parameter controls the up and down motion of the disk for corresponding positive and negative values of S. With an increasing positive value of S, the drag force near the disk increases. Physically, this points to the reality that the frictional forces increase due to the expansion of the disk. The heat transfer rate is also triggered due to the energy transfer process among the accelerating nanoparticles of the hybrid nanofluid. Similarly, the mass transfer process during the reaction process also accelerates for increasing values of S.

(46) Re1/2Cf=A[(f′(0))2+(g(0))2]12,Re−1/2Nu=−C1Dθ′(0),Re−1/2Sh=−ϕ′(0).

Table 4 was constructed to depict the numerical results of the heat transfer rate obtained for various values of the variable wall temperature parameter and the oscillating parameter of the disk. It is seen that for both parameters, the heat transfer rate is enhanced. For a zero value of α, the wall is at a fixed temperature. However, with an increase in α, wall temperature is raised and the heat transfer process increases. The oscillating parameter modifies the impact of the variable wall temperature parameter.

Table 5 quantifies the effect of the upward and downward motion of the disk for surface drag force and the heat transfer rate by fixing the disk rotation to Ω=0.5. For S<0, acceleration of the disk results in a reduction in the drag force. For S=0, the disk is fixed at its position. For S>0, with an increase in S, the decelerating disk slows down the nanoparticles, thus causing the momentum transfer process to increase. Thus, the drag force increases rapidly. The heat transfer rate declines for an accelerating disk (S<0) and increases slowly for a decelerating disk (S>0).

### 4.6. Heat Transfer Rate

The heat transfer rate for the hybrid nanofluid is given by:

(47) Re−1/2Nu=−C1Dθ′(0),

where:

(48) $$\begin{array}{l} C_{1}=\frac{k_{hnf}}{k_{bf}}=\frac{k_{s_{2}}-k_{bf}(1-n)+(1-n)\phi_{2}\left(k_{bf}-k_{s_{2}}\right)}{k_{s_{2}}-(1-n)k_{bf}+\phi_{2}\left(k_{bf}-k_{s_{2}}\right)},\\ D=\frac{k_{bf}}{k_{f}}=\frac{k_{s_{1}}-(1-n)k_{f}+(1-n)\phi_{1}\left(k_{f}-k_{s_{1}}\right)}{k_{s_{1}}-(1-n)k_{f}-\phi_{1}\left(k_{f}-k_{s_{1}}\right)},n=\frac{3}{\psi }, \end{array}$$

Table 6 validates the results presented here by comparing them with those of Turkyilmazoglu et al. [26] for various values of S. In this table, we have considered $\phi_{1}=0,\phi_{2}=0$ for the limiting case. For more visibility, the equations are written.

## 5. Conclusions

The present investigation explored unsteady hybrid nanofluid flow due to a fluctuating spinning disk. Nanoparticles of $Al_{2}O_{3}$ were chosen to detect the best shape for thermal conductivity to obtain an efficient heat transfer process. The physical structure was captured in the form of graphical and tabulated results. The results and discussion section led us to the following significant outcomes.

- Blade-shaped nanoparticles of $Al_{2}O_{3}$ are the best choice for manufacturing the hybrid nanofluid.
- A fluctuating spinning disk inhibits the reaction rate.
- The wall temperature and disk fluctuation parameters increase the heat transfer rate.
- The surface catalysis parameter significantly boosts the reaction rate.
- An increase in the nanoparticle volume fraction boosts the axial velocity profile.

## Figures and Tables

**Table 1:** Thermophysical characteristics of $C_{2}H_{6}O_{2}$, $Al_{2}O_{3}$, and CuO [2,36].

Properties	CuO	$C_{2}H_{6}O_{2}$	$Al_{2}O_{3}$
$k\;(\frac{W}{mK})$	18	0.249	40
$\rho \;(\frac{kg}{m^{3}})$	6500	1116.6	3970
$C_{p}\;\left(JK^{-1}kg^{-1}\right)$	540	2382	765

**Table 2:** Sphericity values for various shapes of the nanoparticles [17,19,36].

Sphericity	Blade	Platelet	Cylinder	Brick	Sphere
ψ	0.36	0.52	0.62	0.81	1.0
**Shape**	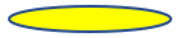	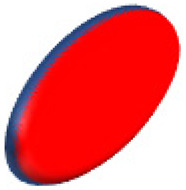	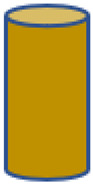	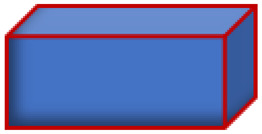	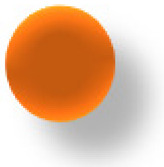

**Table 3:** Numerical outcomes for drag force and the heat and mass transfer rate with disk fluctuation, keeping [26] $K_{vs}=0.5,\lambda =0.5,\Omega =0.1,Sc=1,\mathrm{Pr}=1,\alpha =0.5,K_{s}=0.5,K_{c}=0.5$.

	S=0.1	S=1	S=5	S=10
Re1/2Cf	0.1172760	0.365540	1.90977	3.529200
Re−1/2Nu	0.0515513	0.052562	0.0613628	0.078571
Re−1/2Sh	0.0000128	0.122310	0.403748	0.450621

**Table 4:** Numerical outcomes for the Nusselt number Re−1/2Nu with a varying wall temperature parameter [26] and fixing $K_{vs}=0.5,\lambda =0.5,\Omega =0.5,Sc=1,\mathrm{Pr}=1,K_{s}=0.5,K_{c}=0.5$.

	α=−2	α=−1	α=0	α=0.2	α=0.3
S=1	0.0783255	0.0686171	0.0581365	0.0559352	0.0548204
S=5	0.1552720	0.1243270	0.0855891	0.0764042	0.0715711

**Table 5:** Numerical outcomes of drag force and the heat transfer rate under disk fluctuation and keeping [26] $K_{vs}=0.5,\lambda =0.5,\Omega =0.5,\mathrm{Pr}=1,\alpha =0.3,K_{s}=0.5,K_{c}=0.5$.

	S=−0.3	S=−0.2	S=0	S=0.5	S=1	S=2
Re1/2Cf	0.4199150	0.357956	1.975870	0.5875330	8.782240	18.17200
Re−1/2Nu	0.0327924	0.0245553	0.0213117	0.0530896	0.0548204	0.058571

**Table 6:** Numerical values of θ′(0) with disk fluctuation, keeping $K_{vs}=0,\lambda =0,\Omega =2,\mathrm{Pr}=1$, $\alpha =0.5,K_{s}=0,K_{c}=0,\phi_{1}=0,\phi_{2}=0$.

	S=−0.3	S=−0.2	S=0	S=0.5	S=1	S=2
[26]	0.6480310	0.6180880	0.5603780	0.4221660	0.291822	0.052820
**Present**	**0.6480312**	**0.6180872**	**0.5603776**	**0.4221657**	**0.291821**	**0.052820**

## Nomenclature

**Symbol**	**Unit**	**Description**	**Symbol**	**Unit**	**Description**
V=(u,v,w)	m/s	Components of velocity	λ	Dimensionless	Porosity parameter
Ω	$s^{-1}$	Angular velocity of the disk	Sc	Dimensionless	Schmidt number
ω	Dimensionless	Constant rotation parameter	$A^{*}$, $B^{*}$	Dimensionless	Chemical species
S	Dimensionless	Parameter for controlling the up/down motion of the disk	$C_{a}$	$mol/m^{3}$	Concentration of $A^{*}$
a(t)	m	Vertical displacement of the disk	$C_{b}$	$mol/m^{3}$	Concentration of $B^{*}$
$\dot{a}(t)$	m/s	Vertical velocity of the disk	$k_{s}$,$k_{c}$	${\left(mol\right)}^{2}/s$	Reaction rates
p	Pa	Pressure	$D_{A^{*}}$, $D_{B^{*}}$	$m^{2}/s$	Variable diffusion coefficient of chemical species $A^{*}$, $B^{*}$
$\rho_{hnf}$	$kg/m^{3}$	Density of the hybrid nanofluid	$\tilde{S}$	$m^{-1}$	Interfacial surface area
$\mu_{hnf}$	$kgm^{-1}s^{-1}$	Dynamic viscosity of the hybrid nanofluid	$K_{s}$	Dimensionless	Homogeneous reaction parameter
$k^{*}$	$m^{2}$	Permeability of the medium	$K_{vs}$	Dimensionless	Surface catalysis parameter
$\mu_{f}$	$kgm^{-1}s^{-1}$	Dynamic viscosity of the fluid	$K_{c}$	Dimensionless	Heterogeneous reaction parameter
$\rho_{f}$	$kg/m^{3}$	Density of the fluid	$C_{p}$	$Jkg^{-1}K^{-1}$	Specific heat
T	K	Temperature	δ	Dimensionless	Ratio of the diffusion coefficient
$T_{\infty }$	K	Ambient temperature	α	Dimensionless	Wall temperature parameter
$T_{w}$	K	Wall temperature	$C_{\infty }$	$mol/m^{3}$	Ambient concentration
Pr	Dimensionless	Prandtl number			
$k_{hnf}$	$Js^{-1}m^{-1}K^{-1}$	Thermal conductivity of the hybrid nanofluid			
$k_{bf}$	$Js^{-1}m^{-1}K^{-1}$	Thermal conductivity of the base fluid			
$k_{f}$	$Js^{-1}m^{-1}K^{-1}$	Thermal conductivity of the fluid			

## Appendix A

The following partial derivatives needed to be utilized in Equations (5)–(11):

(A1) $$\begin{array}{l} \frac{\partial u}{\partial t}=\frac{-2r\upsilon f}{a^{3}(t)}-\frac{r\upsilon f\prime }{a^{3}(t)}\dot{a}(t)\left(\eta +1\right),\frac{\partial u}{\partial r}=\frac{\upsilon f}{a^{2}(t)},\frac{\partial u}{\partial z}=\frac{r\upsilon f\prime }{a^{3}(t)},\frac{\partial p}{\partial r}=0,\\ \frac{\partial^{2}u}{\partial r^{2}}=0,\frac{\partial^{2}u}{\partial z^{2}}=\frac{r\upsilon f\prime \prime }{a^{4}(t)}, \end{array}$$

(A2) $$\frac{\partial v}{\partial t}=\frac{-2r\upsilon g}{a^{3}(t)}\dot{a}(t)-\frac{r\upsilon g\prime }{a^{3}(t)}\dot{a}(t)\left(\eta +1\right),\frac{\partial v}{\partial r}=\frac{\upsilon g}{a^{2}(t)},\frac{\partial v}{\partial z}=\frac{r\upsilon g\prime }{a^{3}(t)},\frac{\partial^{2}v}{\partial r^{2}}=0,\frac{\partial^{2}v}{\partial z^{2}}=\frac{r\upsilon g\prime \prime }{a^{4}(t)},$$

(A3) $$\frac{\partial w}{\partial t}=-\frac{\upsilon H\dot{a}(t)}{a^{2}(t)}-\frac{\upsilon H\prime }{a^{2}(t)}\dot{a}(t)\left(\eta +1\right),\frac{\partial w}{\partial r}=0,\frac{\partial w}{\partial z}=\frac{\upsilon H\prime }{a^{2}(t)},\frac{\partial^{2}w}{\partial r^{2}}=0,\frac{\partial^{2}w}{\partial z^{2}}=\frac{\upsilon H\prime \prime }{a^{3}(t)},$$

(A4) $$\begin{array}{l} \frac{\partial T}{\partial t}=-2\alpha c\dot{a}(t){\left(a(t)\right)}^{-2\alpha -1}\left[\alpha \theta +\frac{\eta +1}{2}\theta \prime \right],\frac{\partial T}{\partial r}=0,\\ \frac{\partial T}{\partial z}=\frac{c{\left(a(t)\right)}^{-2\alpha }}{a(t)}\theta \prime ,\frac{\partial^{2}T}{\partial r^{2}}=0,\frac{\partial^{2}T}{\partial z^{2}}=\frac{c{\left(a(t)\right)}^{-2\alpha }}{a^{2}(t)}\theta \prime \prime , \end{array}$$

(A5) $$\frac{\partial C_{a}}{\partial z}=\frac{C_{\infty }\phi \prime }{a(t)},\frac{\partial C_{b}}{\partial z}=\frac{C_{\infty }\xi \prime }{a(t)},\frac{\partial C_{a}}{\partial t}=\frac{C_{\infty }\phi \prime \dot{a}(t)\left(\eta +1\right)}{a^{2}(t)},\frac{\partial C_{b}}{\partial t}=\frac{C_{\infty }\xi \prime \dot{a}(t)\left(\eta +1\right)}{a^{2}(t)},$$

On substituting the above partial derivatives, we can obtain the transformed mathematical model.
